# WRN helicase addiction in MSI-H colorectal cancer: biomarker-guided pharmacology, resistance evolution, and implications for other gastrointestinal cancers

**DOI:** 10.3389/fphar.2026.1962228

**Published:** 2026-10-12

**Authors:** Peng Zhang, Jiangsu Gan, Hongying Wang, Yali Xiong

**Affiliations:** Department of Gastrointestinal Thyroid and Breastsurgery, Chongqing University Affiliated Qianjiang Hospital, Chongqing, China

**Keywords:** biomarker-guided therapy, gastrointestinal cancer, microsatellite instability-high, synthetic lethality, werner syndrome helicase, WRN dependency

## Abstract

Microsatellite instability-high (MSI-H) and mismatch repair-deficient (dMMR) cancers are enriched for dependence on Werner syndrome RecQ helicase (WRN), a synthetic-lethal vulnerability mechanistically linked to expanded TA-dinucleotide repeats, non-B DNA structures, and replication-associated genome instability. However, WRN dependence is not universal among dMMR/MSI-H tumors, indicating that MSI status is an enrichment biomarker rather than a complete predictor of biological dependency. In this review, we synthesize recent advances in WRN structural and molecular pharmacology, with particular emphasis on colorectal cancer, where the strongest functional and pharmacological evidence currently exists. We distinguish four translational biomarker layers: pretreatment predictors of WRN dependency, target-engagement and DNA-damage pharmacodynamic markers, early treatment-response biomarkers, and acquired-resistance markers. We further examine reversible and covalent WRN inhibitors, mechanisms of primary and therapy-emergent resistance, immune and replication-stress-based combinations, and the expanding early-phase clinical landscape. Rather than proposing quantitative WRN dependency as a new biological concept, we present an evidence-graded pharmacological framework that links established substrate biology with drug exposure, target engagement, downstream DNA damage, treatment response, and resistance evolution. This framework may guide prospective biomarker development in MSI-H/dMMR colorectal cancer while identifying the evidence still required for broader application across other gastrointestinal malignancies.

## 1 Introduction

Microsatellite instability-high (MSI-H) and mismatch repair-deficient (dMMR) tumors constitute molecularly distinct subsets of gastrointestinal malignancies characterized by genomic instability, frameshift mutations, increased neoantigen generation, and frequently enhanced immune-cell infiltration (Cancer Genome Atlas, 2012; Cortes-Ciriano et al., 2017; Cancer Genome Atlas Research, 2014). These biological features underlie the marked sensitivity of many MSI-H/dMMR tumors to immune-checkpoint blockade. Durable responses to PD-1 inhibition were first established in dMMR/MSI-H colorectal cancer (CRC) and subsequently demonstrated across multiple tumor types (Le et al., 2015; Le et al., 2017; Le et al., 2020; Andre et al., 2020). PD-1-based regimens, alone or in combination with CTLA-4 blockade, have since produced substantial clinical benefit in metastatic CRC and selected localized colorectal and gastric/gastroesophageal cancers (Overman et al., 2017; Lenz et al., 2022; Andre et al., 2024; Andre et al., 2023; Cercek et al., 2022).

The prevalence of MSI-H/dMMR, however, varies substantially across gastrointestinal malignancies. Approximately 10%–15% of sporadic colorectal cancers are dMMR/MSI-H (Cancer Genome Atlas, 2012), whereas The Cancer Genome Atlas identified an MSI molecular subtype comprising approximately one-fifth of gastric adenocarcinomas (Cancer Genome Atlas Research, 2014). Pan-cancer analyses indicate substantially lower frequencies in many other gastrointestinal malignancies (Cortes-Ciriano et al., 2017). These differences are important when defining the addressable population for WRN-directed therapy and argue against treating MSI-H gastrointestinal cancers as a biologically uniform group. Moreover, MSI-H status does not guarantee durable benefit from immune-checkpoint blockade; primary resistance, incomplete tumor regression, and acquired progression remain clinically relevant and may involve defective antigen presentation, altered interferon signaling, immune exclusion, intratumoral heterogeneity, and lineage-specific oncogenic programs (Kwon et al., 2021). These limitations provide a rationale for therapeutic strategies that exploit vulnerabilities intrinsic to mismatch-repair deficiency rather than relying exclusively on immune activation. Representative pharmacological strategies for targeting WRN and selected clinical-stage WRN inhibitor programs are summarized in Tables 1, 2, respectively. 

**TABLE 1:** Representative pharmacological strategies for targeting WRN in MSI-H cancers.

Agent/modality	Pharmacological class	Binding mechanism/site	Representative quantitative pharmacology	Key pharmacological feature	Development stage	References
HRO761	Reversible allosteric inhibitor	D1–D2 interface; twisted inactive state	WRN ATPase IC50 ≈ 100 nM; SW48 GI50 ≈ 40 nM	Cellular target engagement with MSI-selective downstream DNA damage	Phase I/Ib; NCT05838768	(Ferretti et al., 2024; Rodriguez Perez et al., 2024)
VVD-133214/RO7589831	Covalent allosteric inhibitor	Cys727; compact nucleotide-associated state	Cellular TE50 ≈ 65 nM; HCT116 GI50 ≈ 66 nM	Persistent covalent occupancy and MSI-selective cytotoxicity	Phase I; NCT06004245	(Baltgalvis et al., 2024; Kikuchi et al., 2025)
GSK_WRN3/GSK_WRN4	Covalent WRN inhibitors	Cys727-associated pocket	Biochemical potency in low-nanomolar range	Active in MSI-H cell lines, organoids and xenografts	Preclinical	(Picco et al., 2024)
KWR095/KWR137	Non-covalent WRN inhibitors	HRO761-related allosteric region	ATPase IC50 ≈ 32 nM/8 nM	MSI-H-selective cellular and xenograft activity	Preclinical	(Moon et al., 2025)
WRN hydrophobic-tagging degrader	Chemical degrader	Cys727-directed ligand	DC50 in low-micromolar range	Pharmacological WRN degradation with MSI-selective downstream effects	Preclinical	(Tao et al., 2025)

**Abbreviations:** MSI-H, microsatellite instability-high; WRN, Werner syndrome RecQ-like helicase.

**TABLE 2:** Selected clinical-stage WRN inhibitor programs.

Agent	Sponsor	Modality	Phase	Status (August 31, 2026)	Population	Trial
HRO761	Novartis	Reversible allosteric WRN inhibitor	I/Ib	Active, not recruiting	MSI-H/dMMR solid tumors	(ClinicalTrials.gov, NCT05838768)
VVD-133214/RO7589831	Vividion/Bayer	Covalent WRN inhibitor	I	Recruiting	MSI/dMMR solid tumors	(ClinicalTrials.gov, NCT06004245)
NDI-219216	Nimbus	WRN inhibitor	I/II	Recruiting	Advanced solid tumors	(ClinicalTrials.gov, NCT06898450)
EIK1005	Eikon	WRN inhibitor	I/II	Recruiting	Advanced solid tumors	(ClinicalTrials.gov, NCT07262619)
AMG 436	Amgen	WRN inhibitor	I/Ib	Recruiting	MSI-H/dMMR solid tumors	(ClinicalTrials.gov, NCT07403721)

Trial phase and recruitment status were verified against ClinicalTrials.gov using the prespecified search cut-off of 31 August 2026.

Large-scale functional-genomic studies established WRN as a selective dependency of MSI-H cancer cells, with CRISPR-Cas9 and RNA-interference screens consistently demonstrating a marked viability defect after WRN loss in MSI-H but not MMR-proficient models (Chan et al., 2019; Behan et al., 2019; Lieb et al., 2019; Kategaya et al., 2019). Subsequent work showed that this vulnerability is retained in dMMR colorectal cancer models resistant to immunotherapy, molecularly targeted therapy, and chemotherapy (Picco et al., 2021). Mechanistically, expanded TA-dinucleotide repeats and the non-B DNA structures generated at these loci constitute major replication-associated substrates that create dependence on WRN helicase activity (van Wietmarschen et al., 2020). Selective pharmacological inhibitors, including reversible allosteric and covalent compounds, have subsequently reproduced this synthetic-lethal phenotype in preclinical MSI models (Ferretti et al., 2024; Baltgalvis et al., 2024; Picco et al., 2024).

Importantly, however, dMMR/MSI-H status does not invariably confer equivalent WRN dependency. Picco et al. demonstrated that WRN dependency was present in the large majority, but not all, of a heterogeneous collection of dMMR colorectal cancer models, with WRN-independent outliers showing relatively limited TA-repeat expansion (Picco et al., 2021). More recently, large-scale CRISPR dependency mapping in patient-derived tumor organoids independently identified heterogeneity in WRN dependency among MSI colorectal models, further supporting the distinction between MSI status and functional WRN dependence (Herranz-Ors et al., 2026).

Accordingly, the contribution of the present review is not to propose quantitative WRN dependency as a new biological concept. Instead, we integrate established substrate biology with emerging structural and clinical pharmacology and organize the evidence into distinct translational layers: pretreatment predictors of biological dependency, pharmacological target-engagement and DNA-damage responses, early treatment-response biomarkers, and mechanisms of acquired resistance. This distinction is essential because TA-repeat burden, WRN occupancy, DNA-damage signaling, ctDNA kinetics, and treatment-emergent WRN mutations answer different biological and pharmacological questions and should not be combined into a single undifferentiated measure of “WRN dependency.” Because the most comprehensive mechanistic and pharmacological datasets currently derive from colorectal cancer, evidence from other gastrointestinal malignancies is discussed as emerging and requires disease-specific validation.

### 1.1 Literature search strategy

This article was developed as a narrative rather than a systematic review. Relevant literature was identified through searches of PubMed/MEDLINE, Web of Science, and Scopus, with ClinicalTrials.gov additionally searched for ongoing clinical studies. The literature search was updated through 31 August 2026. Search terms were used individually and in combination and included “WRN,” “Werner helicase,” “microsatellite instability,” “MSI-H,” “mismatch repair deficiency,” “dMMR,” “TA repeat,” “synthetic lethality,” “WRN inhibitor,” “HRO761,” “VVD-133214,” “RO7589831,” “resistance,” “pharmacokinetics,” “pharmacodynamics,” and “clinical trial.” Priority was given to primary functional-genomic, biochemical, structural, medicinal-chemistry, pharmacological, organoid, xenograft, and clinical studies. Recent reviews were used primarily to identify additional primary literature and provide broader context. Reference lists of key publications were manually screened for additional relevant studies. Because the objective was to provide a mechanistically focused narrative synthesis rather than an exhaustive systematic review, no formal PRISMA-based selection process or risk-of-bias assessment was performed. Evidence was interpreted according to experimental level, distinguishing biochemical, genetic, pharmacological, organoid, *in vivo*, and clinical findings wherever possible.

## 2 Molecular basis of WRN dependency

### 2.1 WRN architecture and genome-maintenance functions

WRN is a 1,432-amino-acid member of the human RecQ helicase family and is encoded by the gene mutated in Werner syndrome, an autosomal-recessive disorder characterized by premature ageing, genomic instability, and increased cancer susceptibility (Yu et al., 1996; Yokote et al., 2017). WRN possesses 3′→5′ DNA helicase activity and, unlike the other major human RecQ helicases, also contains an intrinsic 3′→5′ exonuclease domain within the same polypeptide (Suzuki et al., 1997; Shen et al., 1998; Kamath-Loeb et al., 1998). Its domain organization comprises an N-terminal exonuclease region, a central ATP-dependent helicase core formed by the D1 and D2 RecA-like domains, an RQC region containing Zn-binding and winged-helix elements, and a C-terminal helicase-and-RNaseD C-terminal (HRDC) domain located downstream of the helicase/RQC architecture (Choudhary et al., 2004; Kitano et al., 2007; Newman et al., 2021). The D1–D2 helicase core is conformationally flexible, a property that is essential for nucleotide-dependent DNA processing and has subsequently proved central to the development of allosteric WRN inhibitors (Choudhary et al., 2004; Kitano et al., 2007; Newman et al., 2021).

WRN functions within larger genome-maintenance networks rather than as an isolated helicase. Replication protein A enhances WRN processivity, while interactions with FEN1, EXO1, Ku70/80, PARP1, and PCNA connect WRN to stalled-fork processing, end resection, non-homologous end joining, base-excision repair, and strand-displacement synthesis (Brosh et al., 1999; Lee et al., 2018; Sharma et al., 2004; Sharma et al., 2003; Li et al., 2004; Rodriguez-Lopez et al., 2003). WRN acts on structurally diverse DNA substrates, including forked duplexes, D-loops, telomeric DNA, and G-quadruplex-containing structures (Orren et al., 2002; Opresko et al., 2005; Kitano et al., 2010; Edwards et al., 2015; Tang et al., 2016). Single-molecule analyses further indicate that productive unwinding reflects coordinated ATP turnover, DNA binding, conformational rearrangement, and directional translocation rather than ATP hydrolysis alone (Wu et al., 2017).

These functions provide a mechanistic basis for WRN recruitment to perturbed replication forks, where it contributes to fork progression and restart and protects nascent DNA from pathological processing (Datta et al., 2021; Iannascoli et al., 2015). Small-molecule WRN inhibition was explored before recognition of the MSI–WRN synthetic-lethal relationship. NSC 19630 provided early chemical evidence that WRN helicase activity could be pharmacologically inhibited and that WRN inhibition increased sensitivity to replication-associated DNA damage (Aggarwal et al., 2011). However, limited selectivity and suboptimal drug-like properties prevented these early compounds from supporting clinical development.

### 2.2 Genetic discovery of MSI-selective WRN addiction

Four complementary functional-genomic studies independently identified WRN as a selective dependency of MSI-H cancer cells (Chan et al., 2019; Behan et al., 2019; Lieb et al., 2019; Kategaya et al., 2019). Across large-scale CRISPR and RNA-interference screens, depletion of WRN produced marked loss-of-viability phenotypes in MSI-H models but little or no effect in microsatellite-stable counterparts. Re-expression experiments further demonstrated that wild-type WRN and exonuclease-deficient variants could restore viability, whereas helicase-inactive variants could not, establishing ATP-dependent helicase activity as the critical function underlying synthetic lethality (Chan et al., 2019; Behan et al., 2019; Lieb et al., 2019; Kategaya et al., 2019).

This dependency is retained after diverse anticancer treatments. WRN depletion suppressed the growth of immunotherapy-refractory and treatment-resistant dMMR colorectal cancer organoids and xenografts, indicating that acquired resistance to checkpoint blockade, EGFR-directed therapy, or cytotoxic chemotherapy does not necessarily eliminate the underlying requirement for WRN-mediated processing of repetitive DNA structures (Picco et al., 2021). This observation is clinically relevant because early WRN-inhibitor trials have predominantly enrolled heavily pretreated patients.

### 2.3 Expanded TA repeats and context-dependent processing of WRN-dependent DNA lesions

Expanded TA-dinucleotide repeats represent the best-supported proximal substrate underlying WRN dependency in MSI-H cancer cells. These tracts can extend beyond the range captured by conventional MSI assays and adopt non-B DNA structures, including cruciform-like configurations, that impede DNA replication (van Wietmarschen et al., 2020). WRN helicase activity facilitates processing of these replication barriers; consequently, genetic or pharmacological WRN inhibition results in persistent replication stress, RPA accumulation, DNA double-strand breaks, G2/M arrest, chromosome fragmentation, micronucleus formation, and eventual loss of proliferative capacity (van Wietmarschen et al., 2020; Ferretti et al., 2024; Baltgalvis et al., 2024; Picco et al., 2024).

The downstream events should, however, be separated into substrate formation, structure processing, DNA-damage signaling, and cell-death execution rather than interpreted as a single universally established pathway. Recent pharmacological CRISPR screening in MSI-H colorectal cancer identified SMARCAL1 as an important modifier of WRN-inhibitor sensitivity (Ma et al., 2026). SMARCAL1 supports cruciform DNA structures and functionally opposes WRN at these lesions; accordingly, SMARCAL1 depletion confers resistance to WRN inhibition, and this effect depends on its ATPase/translocase activity (Ma et al., 2026). These findings position SMARCAL1 primarily at the level of formation or stabilization of the DNA substrate that creates WRN dependency.

Lesion processing represents a distinct mechanistic layer. Ma et al. identified the MRE11–RAD50–NBS1 (MRN) complex, rather than MUS81 or ERCC1/XPF, as a major mediator of DNA processing following pharmacological WRN inhibition in MSI-H colorectal models (Ma et al., 2026). Disruption of MRN produced profound resistance to WRN inhibition, whereas loss of ATM generated a more modest phenotype, indicating that MRN contributes through both lesion processing and downstream signaling. Importantly, the findings were reproduced using the structurally distinct clinical-stage inhibitor VVD-214, supporting a mechanism that is not restricted to HRO761 (Ma et al., 2026).

Cell-death execution is more context dependent. Genetic experiments in defined colon-cancer models demonstrated that p53 and PUMA can mediate apoptotic responses following WRN loss (Hao et al., 2022). Nevertheless, pharmacological WRN inhibition remains active in TP53-mutant or TP53-deficient MSI models (Ferretti et al., 2024), indicating that p53/PUMA signaling modifies the magnitude or mode of response rather than constituting a universal prerequisite. Time-resolved lineage-tracing further indicates that WRN-deficient cells can undergo heterogeneous fates over multiple cell divisions, including abnormal proliferation, prolonged arrest, senescence-like states, and eventual cell death (Zong et al., 2023). ATR-mediated replication-stress tolerance represents another distinct modifier: partial impairment of WRN and ATR can cooperate, whereas complete disruption of both activities may generate more complex cell-cycle consequences (Zong et al., 2023; Wang et al., 2023).

Together, these observations support a layered model of WRN dependency. Expanded TA-repeat burden determines the availability of a disease-specific replication substrate; SMARCAL1 influences formation or persistence of relevant DNA structures; MRN and ATM contribute to lesion processing and damage signaling; and p53/PUMA, ATR, and additional pathways influence downstream cellular fate (van Wietmarschen et al., 2020; Ferretti et al., 2024; Ma et al., 2026; Hao et al., 2022; Zong et al., 2023; Wang et al., 2023). Importantly, most of these mechanistic relationships have been established in colorectal cancer models and should not automatically be generalized to all MSI-H gastrointestinal malignancies. These mechanistic determinants of WRN dependency and response to WRN inhibition are summarized in Figure 1. 

**FIGURE 1:**
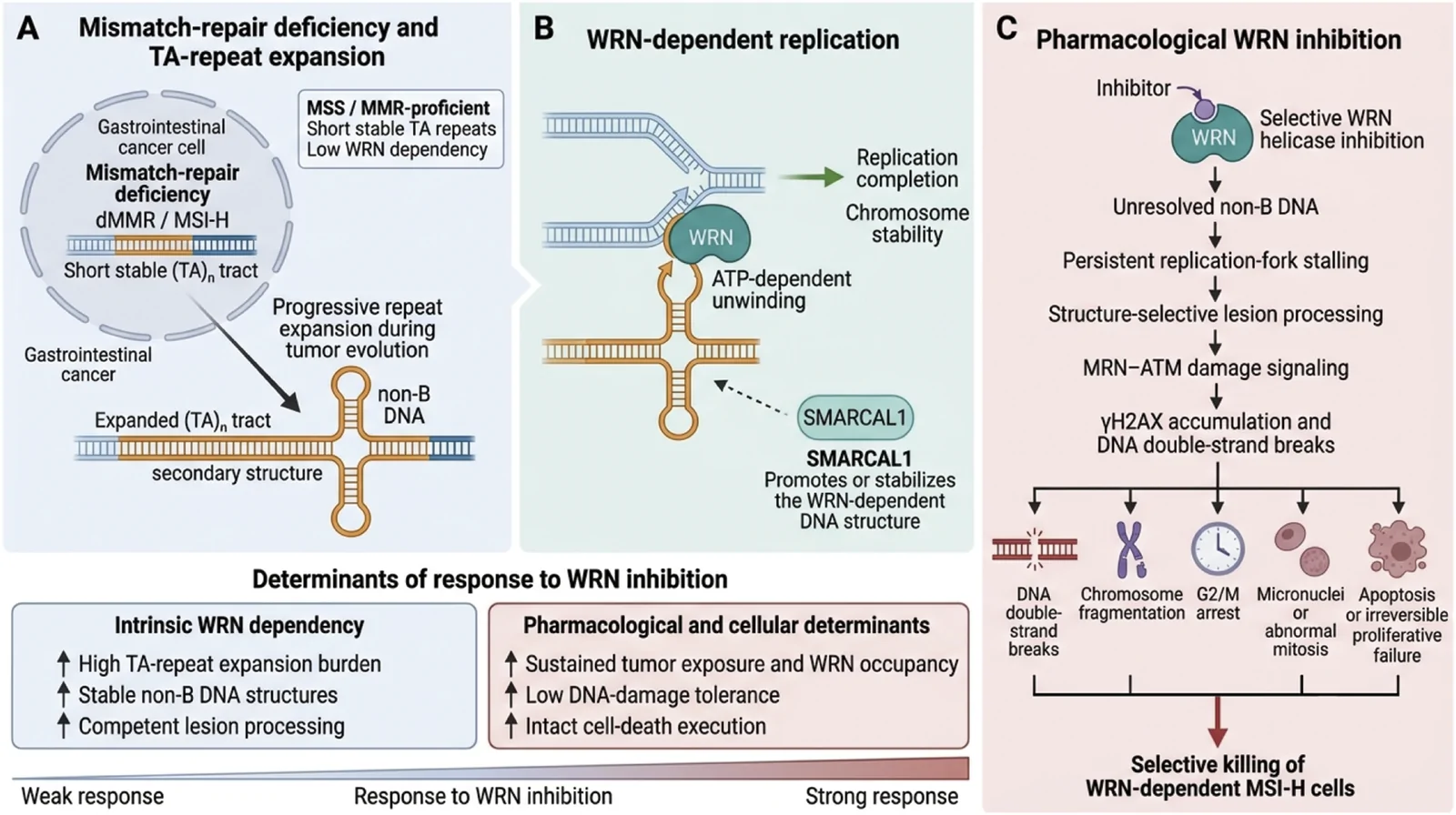
Molecular basis and determinants of WRN dependency in MSI-H colorectal cancer **(A)** Mismatch-repair deficiency promotes expansion of TA-repeat tracts and formation of replication-blocking non-B DNA structures, whereas MMR-proficient tumors generally retain shorter repeats and lower WRN dependency. **(B)** WRN helicase resolves these abnormal DNA structures during replication and thereby supports fork progression and chromosome stability. SMARCAL1 is shown as a modifier that promotes or stabilizes WRN-dependent DNA structures. **(C)** WRN inhibition causes persistent fork stalling, structure-selective lesion processing, MRN–ATM signaling, DNA double-strand breaks, chromosome fragmentation, mitotic abnormalities, and cell death. Therapeutic response is jointly determined by intrinsic WRN dependency—including TA-repeat burden and lesion-processing competence—and pharmacological or cellular factors, including tumor exposure, WRN occupancy, DNA-damage tolerance, and cell-death execution. Created in BioRender. Xiong, Y. (2026) https://BioRender.com/heyqcvs.

## 3 Molecular and structural pharmacology of therapeutics targeting WRN

### 3.1 Allosteric cavities governed by conformational dynamics

The WRN helicase core is conformationally dynamic, with substantial relative mobility between the D1 and D2 RecA-like domains. Nucleotide binding, ATP hydrolysis, DNA engagement, translocation, and product release are coupled to rearrangements of the helicase core that reposition Walker motifs, the arginine finger, and other catalytic elements (Newman et al., 2021; Fletcher et al., 2026). Structural analyses of apo-, nucleotide-, DNA-, and inhibitor-bound WRN indicate that the protein samples multiple conformational states rather than a simple binary open–closed transition (Fletcher et al., 2026).

This conformational plasticity creates transient allosteric pockets that are not apparent from a single static structure. Such sites are pharmacologically attractive because they can avoid direct competition with millimolar intracellular ATP and can stabilize nonproductive WRN conformations in which nucleotide engagement is uncoupled from DNA translocation and duplex unwinding (Ferretti et al., 2024; Baltgalvis et al., 2024; Fletcher et al., 2026). The marked structural differences among these states also provide opportunities for chemical selectivity within the RecQ helicase family.

### 3.2 HRO761 reversibly stabilizes a twisted inactive conformation

HRO761 was identified through high-throughput screening followed by cellular counterselection and structure-guided medicinal-chemistry optimization (Ferretti et al., 2024). The compound binds a non-conserved allosteric pocket at the interface between the D1 and D2 helicase domains and stabilizes a markedly twisted inactive conformation of WRN (Ferretti et al., 2024). Structural comparison with nucleotide-bound WRN demonstrates a major reorientation of the helicase domains, approximately corresponding to a 180° rotation of D2 relative to D1, that disrupts the geometry required for productive ATPase-driven DNA unwinding (Ferretti et al., 2024; Fletcher et al., 2026). The binding site is unusually polar and arginine-rich and partly overlaps structural elements involved in the D2 ATP half-site, allowing HRO761 to inhibit helicase activity without acting as a conventional ATP-competitive inhibitor (Ferretti et al., 2024).

#### 3.2.1 Target engagement is distinct from biological response

Target engagement should be distinguished from biological dependency and downstream cytotoxicity. HRO761 inhibits WRN ATPase activity with a biochemical IC50 of approximately 100 nM under high-ATP assay conditions, which translates to a 4-day GI50 of approximately 40 nM in SW48 MSI cells (Ferretti et al., 2024). Across a broader panel, MSI cell lines showed HRO761 GI50 values of approximately 50–1,000 nM in longer-term clonogenic assays, whereas MSS models remained largely insensitive (Ferretti et al., 2024). In contrast, cellular WRN target engagement occurred in both MSI and MSS models, with half-maximal protein-stabilization values generally within the 10–100 nM range (Ferretti et al., 2024). These findings demonstrate that ligand binding is necessary but insufficient for cytotoxicity: productive pharmacological response additionally requires a tumor context in which loss of WRN activity is converted into persistent replication-associated DNA damage.

In WRN-dependent cells, HRO761 treatment induces ATM/CHK2 signaling, γH2AX accumulation, G2 arrest, chromatin retention of WRN, and context-dependent WRN degradation (Ferretti et al., 2024; Rodriguez Perez et al., 2024). The latter involves PIAS4-dependent SUMOylation, RNF4 recruitment, and p97/VCP-mediated extraction of chromatin-associated WRN (Rodriguez Perez et al., 2024). Therefore, total WRN abundance, ligand occupancy, chromatin-associated WRN, and downstream DNA-damage markers represent related but mechanistically distinct pharmacodynamic measurements.

### 3.3 VVD-133214/RO7589831 mediates nucleotide-assisted covalent blockade

VVD-133214, also termed VVD-214 and subsequently developed clinically as RO7589831, emerged from covalent chemoproteomic discovery efforts (Baltgalvis et al., 2024; Kikuchi et al., 2025). The compound covalently engages Cys727 near the D1–D2 interface in a nucleotide-facilitated manner and stabilizes a compact inactive WRN conformation that lacks the conformational flexibility required for productive helicase activity (Baltgalvis et al., 2024). Covalent engagement allows target occupancy to persist after free-drug concentrations decline, creating a pharmacological profile distinct from that of reversible inhibitors such as HRO761 (Baltgalvis et al., 2024; Kikuchi et al., 2025).

For covalent inhibitors, dose, concentration, and systemic exposure remain fundamental pharmacological variables. Their pharmacological effect, however, is additionally determined by the kinetics of covalent target capture, tissue exposure, fractional target occupancy, persistence of occupancy after free-drug clearance, and synthesis of replacement WRN (Baltgalvis et al., 2024; Kikuchi et al., 2025). Consequently, equilibrium IC50 values alone incompletely characterize irreversible WRN inhibitors and should, where available, be interpreted together with time-dependent target-engagement measurements, covalent-inactivation efficiency, and target-resynthesis kinetics (Baltgalvis et al., 2024; Kikuchi et al., 2025).

Cys727 therefore represents a productive covalent handle, and chemically distinct electrophile-bearing scaffolds demonstrate that the inducible WRN pocket can accommodate substantial chemical diversity (Parker et al., 2023; Tong et al., 2025). However, scaffold diversity should not be equated with resistance orthogonality. Compounds that retain dependence on Cys727 engagement or on overlapping inhibitor-accessible conformations may remain susceptible to shared on-target resistance mechanisms. Thus, true pharmacological orthogonality should be established functionally across resistance-associated WRN variants rather than inferred from chemical scaffold dissimilarity alone.

### 3.4 Expanding the accessible chemical space

Several parallel medicinal-chemistry programs have expanded the chemical space available for WRN inhibition. Triazolo-pyrimidine derivatives retained biochemical activity and MSI-selective cellular effects while offering substantial opportunities for optimization of physicochemical properties (Sui et al., 2025). Bioisosteric replacement generated KWR095 and related non-covalent compounds with activity in MSI-H colorectal cancer cells and xenograft models (Moon et al., 2025). Independent ATPase-based screening programs have also identified water-soluble spirocyclic inhibitors with preliminary cellular selectivity (Xu et al., 2025). Additional thiophen-2-ylmethylene bis-dimedone compounds and virtual-screening-derived ATP-site ligands have been reported, although their selectivity, permeability, tumor exposure, and resistance profiles remain less comprehensively defined (Yang et al., 2024; Yuan et al., 2024).

Within covalent series, structure-guided optimization has improved pharmacokinetic exposure while maintaining productive engagement of the Cys727-associated pocket (Xu et al., 2026). Future comparison of WRN chemotypes should therefore extend beyond potency against wild-type WRN and include standardized assessment across resistance-associated WRN variants, other RecQ helicases, relevant DNA substrates, cellular target engagement, and *in vivo* exposure.

### 3.5 Catalytic blockade, molecular trapping, and protein removal

Pharmacological targeting of WRN can produce three mechanistically distinct but potentially overlapping outcomes: catalytic inhibition, stabilization of nonproductive conformational states, and reduction of cellular WRN abundance. Catalytic inhibition directly suppresses ATP-dependent helicase activity, whereas conformational trapping stabilizes a WRN state that cannot support productive DNA processing. Protein degradation represents a third consequence by reducing the total pool of WRN available for either catalytic or scaffolding functions. HRO761 and VVD-133214 primarily inhibit helicase activity by stabilizing inactive conformations, whereas changes in chromatin association and protein abundance arise secondarily and in a cellular-context-dependent manner (Ferretti et al., 2024; Baltgalvis et al., 2024; Rodriguez Perez et al., 2024).

Conditional degron experiments demonstrate that acute WRN depletion recapitulates chromosome breakage and G2/M arrest selectively in MSI-H cells (Tejwani et al., 2024). Ligand-directed covalent hydrophobic tagging has further provided chemical proof of principle that WRN can be pharmacologically eliminated (Tao et al., 2025). Such event-driven approaches could, in principle, overcome resistance caused by increased WRN abundance; however, degraders derived from first-generation WRN-binding ligands may remain vulnerable to binding-site mutations and may additionally face limitations related to permeability, tissue distribution, E3-ligase availability, and ternary-complex formation.

Earlier evidence of MDM2-dependent WRN degradation following DNA damage further emphasizes that total WRN abundance is dynamically regulated and cannot be assumed to report either transcriptional output or target engagement directly (Liu et al., 2019). Clinical pharmacodynamic studies should therefore distinguish total WRN, ligand-occupied WRN, chromatin-associated WRN, and downstream DNA-damage responses.

### 3.6 Translational requirements for pharmacokinetics and pharmacodynamics

For reversible inhibitors, pharmacological activity is expected to require sufficient unbound intratumoral exposure to maintain productive WRN inhibition across multiple rounds of DNA replication. Covalent inhibitors are additionally governed by target-capture kinetics, occupancy persistence, and synthesis of replacement WRN (Ferretti et al., 2024; Baltgalvis et al., 2024; Kikuchi et al., 2025). In either setting, biochemical potency alone is insufficient unless target inhibition is translated into sustained replication-associated DNA damage.

A translational PK/PD framework should therefore evaluate, in sequence, systemic and intratumoral exposure, direct target engagement or occupancy, chromatin trapping or WRN depletion when relevant, and downstream DNA-damage responses such as γH2AX, pKAP1, pCHK2, and p21 (Ferretti et al., 2024; Rodriguez Perez et al., 2024; Kikuchi et al., 2025). ctDNA decline, durable disease stabilization, and radiographic tumor regression should be interpreted as downstream response measures rather than evidence of target engagement. This separation is essential for distinguishing inadequate drug delivery from insufficient biological dependency or therapy-emergent escape. The conformation-selective pharmacology, cellular consequences, resistance mechanisms, and integrated PK/PD framework for WRN‐directed therapeutics are summarized in Figure 2.

**FIGURE 2:**
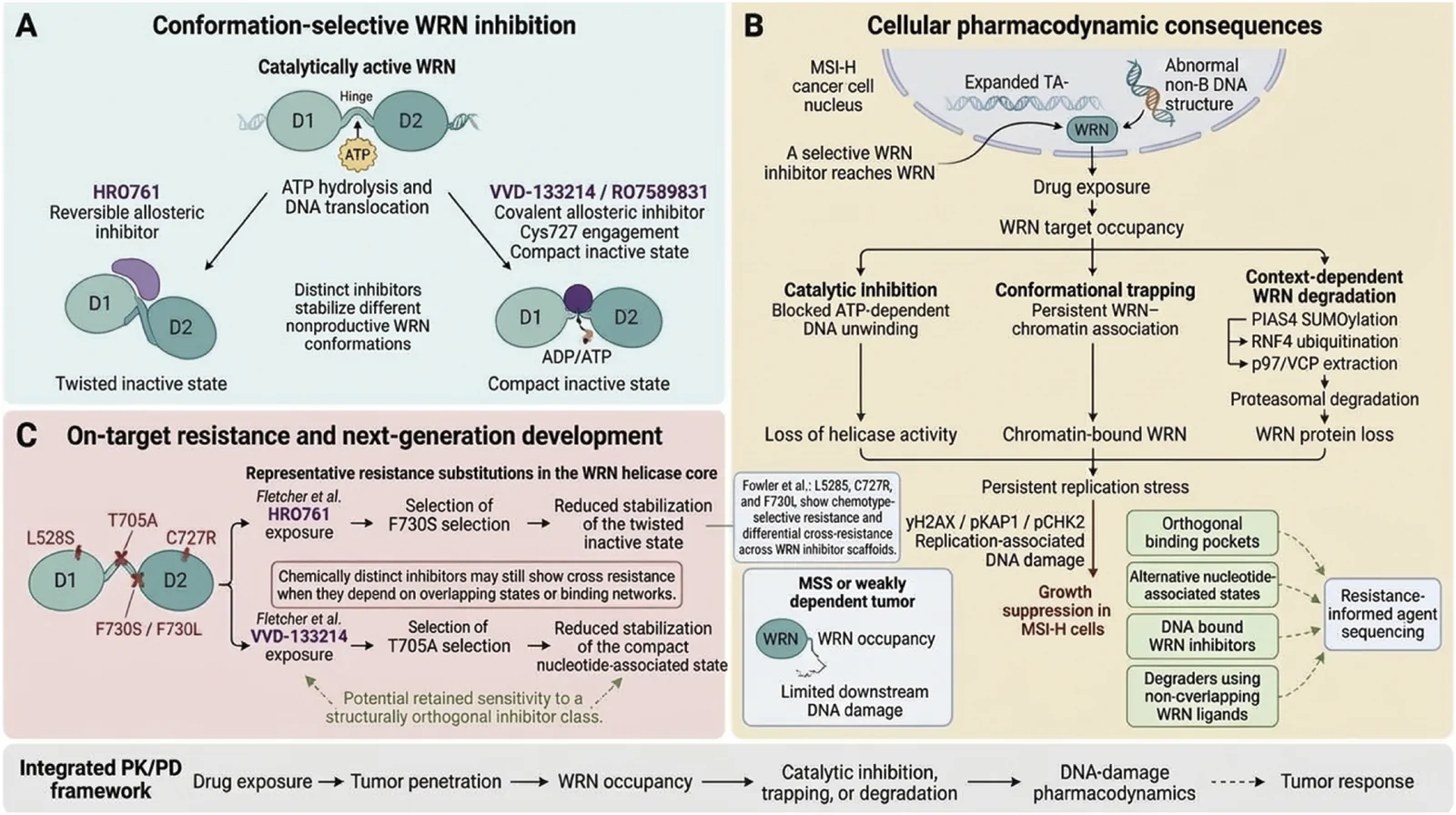
Conformation-selective pharmacology and resistance-informed development of WRN-directed therapeutics **(A)** HRO761 and VVD-133214/RO7589831 stabilize distinct nonproductive WRN conformations. HRO761 acts as a reversible allosteric inhibitor that favors a twisted inactive state, whereas VVD-133214 covalently engages Cys727 and stabilizes a compact nucleotide-associated state. **(B)** Target occupancy can produce catalytic inhibition, chromatin trapping, and context-dependent WRN degradation through the PIAS4–RNF4–p97/VCP pathway. These effects converge on replication stress and DNA-damage signaling in WRN-dependent MSI-H cells, whereas weakly dependent tumors may show occupancy without substantial downstream damage. **(C)** Resistance-associated substitutions include L528S, T705A, C727R, F730S, and F730L. Fletcher et al. identified F730S after HRO761 exposure and T705A after VVD-133214 exposure, whereas Fowler et al. reported chemotype-selective L528S, C727R, and F730L variants. These differential resistance profiles support development of structurally orthogonal inhibitors, DNA-bound-state ligands, and degraders using non-overlapping WRN-binding elements. Created in BioRender. Xiong, Y. (2026) https://BioRender.com/wq0e296.

## 4 Biomarkers for patient selection, pharmacodynamic assessment, and longitudinal monitoring

Biomarkers in WRN-directed therapy should be classified according to their biological and clinical purpose rather than combined into a single measure of “WRN dependency.” Pretreatment predictors include MMR/MSI status and expanded TA-repeat burden; candidate functional modifiers include factors governing formation and processing of WRN-dependent DNA structures. WRN occupancy, chromatin-associated WRN, and DNA-damage signals represent target-engagement or pharmacodynamic measurements. By contrast, ctDNA decline is a downstream treatment-response measure, whereas emergent WRN mutations and changes in WRN abundance represent resistance-monitoring biomarkers (van Wietmarschen et al., 2020; Ferretti et al., 2024; Baltgalvis et al., 2024; Picco et al., 2024; Hao et al., 2022; Rodriguez Perez et al., 2024; Zou et al., 2020; Emiloju et al., 2024; Ryoo et al., 2023; Osumi et al., 2019; Malla et al., 2022; Roazzi et al., 2024; Fowler et al., 2026).

### 4.1 MMR/MSI status as a pretreatment enrichment biomarker

Established approaches for identifying dMMR/MSI-H gastrointestinal cancers include immunohistochemistry for mismatch-repair proteins, PCR-based microsatellite panels, next-generation sequencing, and computational methods such as MSIsensor, MSIsensor-pro, and MSIcall (Niu et al., 2014; Jia et al., 2020; Zhu et al., 2018; Pabla et al., 2019; Hirotsu et al., 2020). These platforms are clinically scalable and therefore provide a practical first step for enrollment into WRN-inhibitor trials.

However, routine MSI assays were developed to classify the molecular consequence of mismatch-repair deficiency rather than to quantify the expanded TA-repeat substrates that underlie WRN dependency (van Wietmarschen et al., 2020; Yakushina et al., 2023). Although immunohistochemistry, PCR-based approaches, and sequencing-based assays show high overall concordance, discrepant cases occur and may require expert pathology review and orthogonal retesting (Guyot D’Asnieres De Salins et al., 2021; Ali-Fehmi et al., 2024). Accordingly, MSI-H/dMMR status should be considered a clinically useful enrichment biomarker rather than a complete companion diagnostic for WRN dependency.

### 4.2 Quantifying expanded TA-repeat burden

Expanded TA-repeat burden represents a candidate pretreatment predictor of biological WRN dependency because it more directly reflects the lesion-generating substrate than conventional genome-wide MSI classification. Large TA-repeat expansions can be resolved directly by long-read sequencing, whereas short-read sequencing may infer expansion through locus-specific coverage abnormalities, alignment disruption, and validated computational signatures (van Wietmarschen et al., 2020). WRN-independent dMMR colorectal cancer models generally show less extensive TA-repeat expansion than strongly WRN-dependent counterparts (Picco et al., 2021), and pharmacological sensitivity to selective WRN inhibitors is likewise associated with expanded TA-repeat burden (Picco et al., 2024).

At present, however, no clinically validated TA-expansion threshold defines eligibility for WRN-inhibitor therapy. We therefore propose a prospective, testable model rather than an arbitrary clinical cut-off: baseline TA-expansion burden should be evaluated as a continuous candidate predictor of biological dependency, whereas pharmacological target engagement and downstream DNA damage should be measured independently. This model predicts that, at comparable intratumoral WRN occupancy, tumors with greater TA-expansion burden will show stronger induction of replication-associated DNA-damage markers and greater early treatment response. Conversely, failure to observe an association between baseline TA-expansion burden and downstream pharmacodynamic or response measurements after controlling for drug exposure and target occupancy would argue against the proposed substrate-response relationship.

### 4.3 Functional modifiers of biological dependency

Expanded TA-repeat burden determines the availability of a relevant DNA substrate, whereas cellular factors influence whether those structures are formed, processed, and converted into lethal lesions. SMARCAL1 promotes or stabilizes cruciform-like DNA structures and its depletion can confer resistance to WRN inhibition (Ma et al., 2026). MRN is required for efficient processing of these lesions, whereas ATM contributes downstream damage signaling but appears to have a more modest effect on drug sensitivity (Ma et al., 2026). p53/PUMA and ATR influence subsequent cell-fate decisions in a context-dependent manner (Hao et al., 2022; Zong et al., 2023; Wang et al., 2023). These factors should therefore be regarded as candidate modifiers of WRN dependence or response rather than components of a single validated companion-diagnostic signature.

### 4.4 Target-engagement and pharmacodynamic biomarkers

Pharmacodynamic assessment addresses a different question from pretreatment prediction: whether a drug has reached and functionally inhibited WRN within the tumor. Direct WRN occupancy or target-engagement assays provide the most proximal measurement, whereas chromatin-associated WRN, WRN depletion, γH2AX, pKAP1, pCHK2, RPA accumulation, p21 induction, and chromosome damage report increasingly downstream pharmacodynamic consequences (Ferretti et al., 2024; Rodriguez Perez et al., 2024). HRO761 provides a clear example of why these measurements must be separated: comparable WRN target engagement can be achieved in MSI and MSS cells, yet persistent DNA-damage signaling and growth inhibition occur predominantly in WRN-dependent MSI contexts (Ferretti et al., 2024). Thus, occupancy without downstream DNA damage should prompt consideration of limited biological dependency or ineffective lesion conversion rather than automatic dose escalation.

### 4.5 Early-response and resistance-monitoring biomarkers

Circulating tumor DNA is well suited to longitudinal monitoring in colorectal cancer. Serial ctDNA measurements can provide early estimates of tumor burden, detect molecular residual disease, and identify treatment-emergent genomic alterations before conventional radiographic progression in selected settings (Zou et al., 2020; Emiloju et al., 2024; Ryoo et al., 2023; Osumi et al., 2019; Malla et al., 2022; Roazzi et al., 2024). In WRN-directed therapy, ctDNA decline should be interpreted as an early molecular response biomarker rather than as evidence of target engagement. Conversely, newly emerging WRN helicase-domain substitutions or changes in WRN expression represent candidate resistance biomarkers (Fletcher et al., 2026; Fowler et al., 2026).

A rational biomarker sequence therefore consists of four distinct layers: first, MMR/MSI and TA-repeat assessment for pretreatment enrichment and prediction; second, exposure and WRN target engagement; third, downstream DNA-damage pharmacodynamics; and fourth, longitudinal assessment of ctDNA response and resistance evolution. No single biomarker is expected to perform all of these functions. A broader evidence-graded translational framework integrating patient enrichment, WRN dependency assessment, pharmacological evaluation, candidate combination strategies, and dynamic monitoring is shown in Figure 3.

**FIGURE 3:**
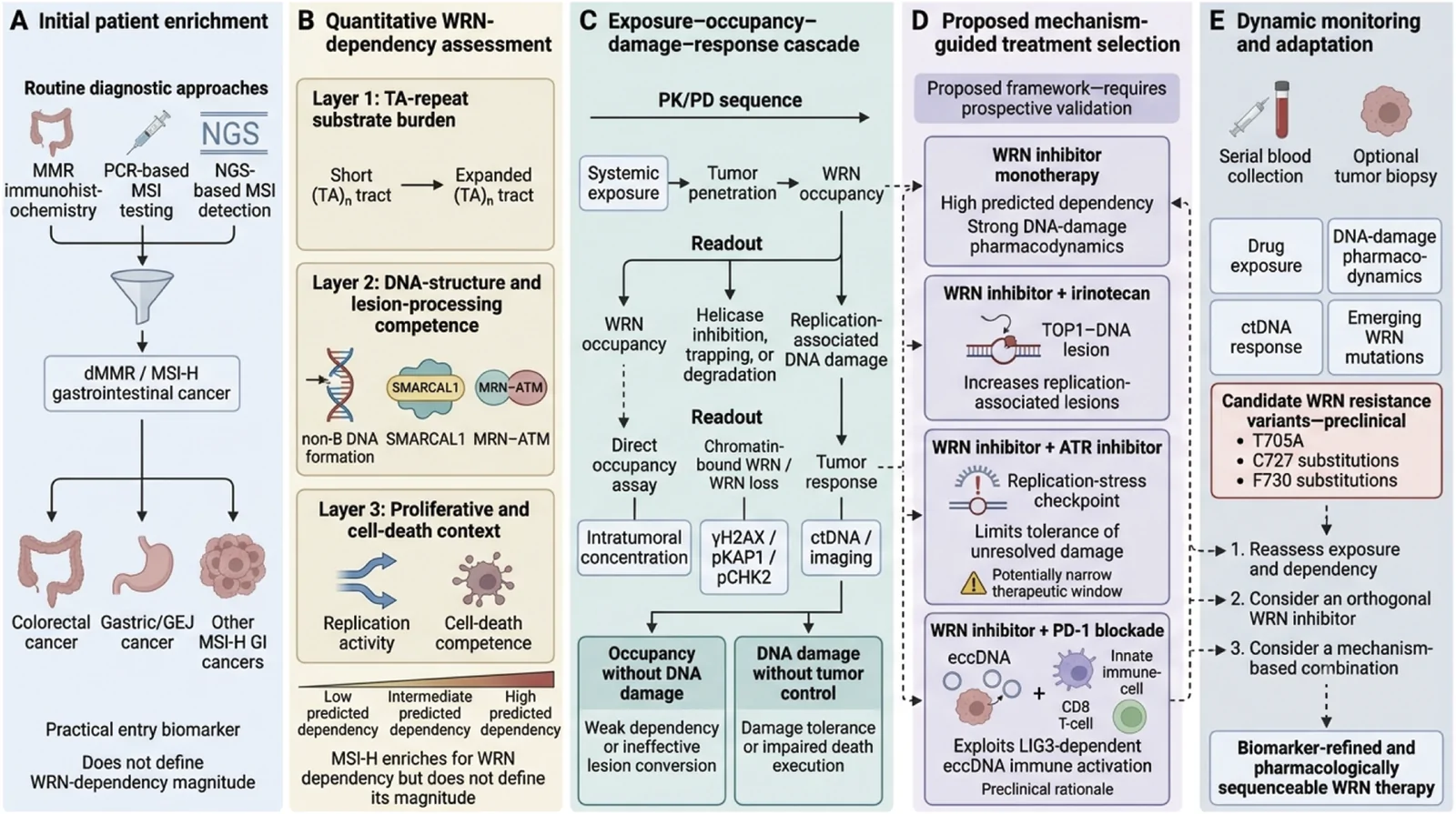
Evidence-graded biomarker and mechanism-informed framework for WRN-directed therapy **(A)** MMR/MSI testing provides initial patient enrichment, while TA-repeat burden and functional modifiers further refine predicted WRN dependency. **(B)** WRN dependency is influenced by DNA-structure formation, lesion processing, replication activity, and cell-death competence. **(C)** Pharmacological assessment separates drug exposure, WRN occupancy, downstream DNA-damage pharmacodynamics, and treatment response. **(D)** Candidate combination strategies are presented according to their current evidence level, including irinotecan, PD-1 blockade, and ATR inhibition; these remain investigational and require prospective validation. **(E)** Serial monitoring of exposure, ctDNA, DNA-damage responses, and emerging WRN alterations may support resistance-informed therapeutic adaptation. Created in BioRender. Xiong, Y. (2026) https://BioRender.com/ktejrq4.

## 5 Intrinsic and therapy-emergent resistance

Intrinsic insensitivity and therapy-emergent resistance should be considered separately because they arise at different stages of treatment and require different interventions. Limited baseline TA-repeat expansion, reduced formation of relevant non-B DNA structures, altered lesion-processing competence, slow proliferation, or inadequate intratumoral drug exposure may reduce apparent sensitivity despite an MSI-H/dMMR genotype (Picco et al., 2021; van Wietmarschen et al., 2020; Picco et al., 2024; Ma et al., 2026). Treatment duration is also relevant because WRN inhibition can require several rounds of replication before sufficient damage accumulates to produce an antiproliferative phenotype (Ferretti et al., 2024; Zong et al., 2023).

Acquired resistance, by contrast, can arise directly through evolution of WRN itself. Fowler et al. demonstrated rapid emergence of clustered helicase-domain mutations during prolonged WRN-inhibitor exposure in MSI models (Fowler et al., 2026). Most resistance substitutions conferred broad cross-resistance across the tested compounds, whereas L528S, C727R, and F730L showed chemotype-selective resistance profiles (Fowler et al., 2026). These findings demonstrate that apparently related WRN inhibitors are not necessarily pharmacologically interchangeable and that some resistant alleles may preserve sensitivity to structurally distinct compounds.

A complementary structural study by Fletcher et al. identified F730S after chronic HRO761 exposure and T705A after chronic VVD-133214 treatment (Fletcher et al., 2026). HRO761-resistant cells displayed more than 500-fold loss of HRO761 sensitivity, whereas the F730S population retained partial sensitivity to VVD-133214 (Fletcher et al., 2026). In VVD-133214-selected cells, T705A disrupted structural interactions that stabilize the inhibitor-associated conformation (Fletcher et al., 2026). Importantly, these findings should be distinguished from the L528S, C727R, and F730L chemotype-selective mutations reported by Fowler et al. (Fowler et al., 2026).

Altered WRN abundance should also be considered a resistance-associated mechanism rather than a purely hypothetical possibility. Fletcher et al. observed greater than twofold WRN upregulation during late stages of chronic inhibitor selection, although the temporal sequence indicated that increased expression alone did not account for early resistance (Fletcher et al., 2026). Fowler et al. likewise identified altered WRN expression alongside point mutations during resistance evolution (Fowler et al., 2026). Increased WRN abundance could decrease fractional occupancy at a given drug exposure or accelerate restoration of an uninhibited target pool, particularly for covalent inhibitors whose pharmacology is influenced by target resynthesis (Fletcher et al., 2026; Kikuchi et al., 2025; Fowler et al., 2026).

A particularly concrete strategy for pharmacological orthogonality is to target a different conformational state of WRN. Structural analyses suggest that both HRO761 and VVD-133214 preferentially stabilize inactive “off-DNA” conformations (Fletcher et al., 2026). Fletcher et al. therefore proposed development of compounds capable of engaging DNA-bound, or “on-DNA,” WRN as a means of accessing a structurally distinct pharmacological state and potentially circumventing resistance centered on first-generation inhibitor-accessible pockets (Fletcher et al., 2026). This strategy remains a structure-guided hypothesis and should be evaluated directly against standardized panels of resistance-associated WRN variants before clinical extrapolation.

Resistance-informed clinical development should combine serial ctDNA profiling with tissue sampling when feasible. Detection of emerging WRN mutations would support an on-target evolutionary mechanism, whereas persistent target occupancy without downstream DNA-damage signaling may indicate reduced functional dependency or altered lesion processing. Conversely, initial pharmacodynamic activation followed by later progression should prompt assessment of WRN mutations, altered WRN abundance, and adaptive changes in downstream damage-response pathways (Ma et al., 2026; Fletcher et al., 2026; Fowler et al., 2026). Mechanism-based interpretation is therefore essential when considering sequencing or substitution of WRN inhibitors with different pharmacological properties.

## 6 Immunological pharmacology and rational combination strategies

WRN inhibition can generate immunological consequences in addition to direct synthetic-lethal tumor-cell killing. In MSI colorectal cancer models, pharmacological WRN inhibition promoted the release of extrachromosomal circular DNA (eccDNA), which was associated with immune-cell activation, cytokine production, and enhanced antitumor immunity (Hao et al., 2026). Importantly, deletion of nuclear LIG3 abolished both the antitumor and immunogenic consequences of WRN inhibition, establishing LIG3-dependent eccDNA generation as a functional component of this response rather than a passive by-product of chromosome damage (Hao et al., 2026). WRN inhibition also enhanced the activity of PD-1 blockade in preclinical MSI colorectal cancer models (Hao et al., 2026).

These findings provide direct WRN-specific evidence linking genome damage with antitumor immunity; however, induction of immunogenic DNA species should not be equated with guaranteed therapeutic immune control. Productive antitumor immunity additionally depends on tumor-antigen visibility, immune editing, intratumoral heterogeneity, immune-cell infiltration, and the broader immunosuppressive tumor microenvironment. These considerations are contextual determinants of immune efficacy rather than direct components of the WRN–eccDNA mechanism (Singh et al., 2026; Roerden and Spranger, 2025).

Because WRN inhibition induces chromosome breakage, micronucleus formation, and immunostimulatory DNA species, cytosolic DNA-sensing pathways such as cGAS–STING provide a plausible mechanistic connection between WRN-induced genome instability and innate immune activation. However, the current WRN-specific evidence more directly establishes LIG3-dependent eccDNA generation and downstream immunogenicity than universal dependence on cGAS–STING (Hao et al., 2026). cGAS–STING should therefore be considered a testable downstream mechanism rather than an established obligatory component of WRN-inhibitor activity.

Combination strategies should be interpreted according to the level of WRN-specific evidence. Direct preclinical pharmacological evidence supports combining HRO761 with irinotecan: subefficacious concentrations of the two agents enhanced antiproliferative activity and DNA damage in MSI cells, and the combination produced complete tumor regression in SW48 xenografts (Ferretti et al., 2024). These experiments also showed acceptable short-term tolerability in mice, although this does not establish a clinical therapeutic window (Ferretti et al., 2024).

Direct mechanistic preclinical evidence also supports combination with PD-1 blockade through LIG3-dependent eccDNA-associated immune activation in MSI colorectal cancer (Hao et al., 2026). By contrast, ATR inhibition represents a mechanistically plausible replication-stress combination supported by genetic and pharmacological interactions between WRN and ATR (Zong et al., 2023; Wang et al., 2023), but the magnitude and safety of this interaction remain incompletely characterized. Moreover, not every DNA-damage-response inhibitor is expected to cooperate with WRN inhibition: disruption of MRN markedly reduces the formation or processing of lethal WRN-dependent lesions and can therefore confer resistance rather than synergy (Ma et al., 2026).

Combination-specific safety will require particular attention because irinotecan and WRN inhibition may produce overlapping gastrointestinal and hematological toxicities, whereas ATR-directed combinations may further reduce replication-stress tolerance in proliferating normal tissues. Clinical dose and schedule optimization should therefore integrate toxicity with exposure and pharmacodynamic measurements.

## 7 Initial clinical translation

HRO761 is an orally administered reversible allosteric WRN inhibitor being evaluated in the phase I/Ib study NCT05838768 in patients with advanced MSI-H/dMMR solid tumors, as monotherapy and in combination with pembrolizumab or irinotecan (Foote et al., 2025; ClinicalTrials.gov). In the updated single-agent dose-escalation analysis presented at the ESMO Congress 2025 (data cut-off, 15 June 2025), 57 patients had received HRO761 across once-daily dose cohorts of 100, 200, 300, 600, and 900 mg and twice-daily dose cohorts of 150, 300, and 450 mg; 33/57 patients (57.9%) had colorectal cancer (CRC). Among 55 patients with a post-baseline tumor assessment, the CRC subgroup comprised 33 patients, of whom 2/33 (6.1%) achieved a partial response and 24/33 (72.7%) had stable disease, corresponding to a disease-control rate of 78.8% (26/33; 95% CI, 61.1–91.0) (Foote et al., 2025).

Median progression-free survival in the CRC cohort (n = 33) was 8.1 months (95% CI, 4.4–not reached), while median PFS had not been reached among the 30 CRC patients treated at total daily doses ≥200 mg at the data cut-off (Foote et al., 2025). An exploratory ctDNA analysis included 20 patients with detectable ctDNA at baseline and an available cycle 2 days 1 sample; 10 patients achieved ctDNA clearance, all of whom had CRC. The presentation summarized this as approximately 70% of evaluable CRC patients with detectable baseline ctDNA achieving clearance after approximately 1 month of treatment (Foote et al., 2025). Because these PFS and ctDNA findings derive from a nonrandomized early-phase dose-escalation cohort, they should be considered exploratory and should not be interpreted as established efficacy, validated surrogate endpoints, or direct evidence of WRN target engagement.

RO7589831/VVD-214 is an irreversible covalent WRN inhibitor being evaluated in the first-in-human phase I study NCT06004245 in patients with MSI/dMMR advanced solid tumors (Baltgalvis et al., 2024; Kikuchi et al., 2025; Yap et al., 2025; ClinicalTrials.gov. NCT06004245). In the primary AACR 2025 presentation (data cut-off, 25 November 2024), 44 patients had been enrolled across six dose cohorts spanning total daily doses of 150–2000 mg, administered once or twice daily; 22 patients had CRC and 22 had non-CRC tumors. First-dose pharmacokinetic analysis was available for 41 patients and showed rapid absorption, with a mean Tmax of 2.6 ± 1.3 h and a plasma half-life of 4.4 ± 1.8 h (Yap et al., 2025).

Among 32 efficacy-evaluable patients with MSI tumors, four RECIST v1.1 partial responses were reported, including two confirmed and two ongoing unconfirmed responses; these occurred in one patient with CRC, one with ovarian cancer, and two with endometrial cancer. Twenty of 32 patients (62.5%) achieved stable disease, corresponding to a disease-control rate of 68.8% (95% CI, 51.13–86.37). Exploratory FDG-PET metabolic responses were observed in 15/31 evaluable patients, while ctDNA molecular responses were observed in 2/2 patients with partial response, 8/9 with stable disease, and 0/5 with progressive disease (Yap et al., 2025). These findings provide preliminary evidence of clinical and pharmacological activity, but the response, imaging, and ctDNA results remain exploratory because they derive from an early-phase dose-escalation study. Direct cross-trial comparison with HRO761 is therefore inappropriate. Moreover, because RO7589831 covalently engages WRN, plasma half-life alone should not be interpreted as the duration of target inhibition; target occupancy and WRN resynthesis remain important determinants of pharmacological persistence (Baltgalvis et al., 2024; Kikuchi et al., 2025).

### 7.1 Safety, tolerability, and potential on-target risk

The relationship between therapeutic WRN inhibition and Werner syndrome requires explicit consideration. Biallelic germline loss of WRN causes a multisystem premature-ageing syndrome characterized by genomic instability and increased cancer susceptibility (Yu et al., 1996; Yokote et al., 2017). Nevertheless, lifelong congenital WRN deficiency should not be considered equivalent to pharmacological inhibition initiated in adults, which is dose dependent, temporally restricted, and incomplete across tissues. Preclinical studies of HRO761 and VVD-133214 demonstrated antitumor activity at tolerated exposures in animal models, supporting the existence of at least an initial therapeutic window (Ferretti et al., 2024; Baltgalvis et al., 2024).

Long-term on-target safety nevertheless remains unresolved. Particular attention should be paid to highly proliferative normal tissues, including hematopoietic and gastrointestinal compartments, and to the possibility of cumulative genome-maintenance effects during prolonged treatment. Early clinical experience with HRO761 and RO7589831 has thus far identified predominantly manageable acute toxicities, but the available studies remain early-phase investigations with limited duration of follow-up. Future trials should therefore integrate conventional adverse-event monitoring with duration of exposure, target occupancy, hematological recovery, gastrointestinal toxicity, and prolonged treatment duration.

Clinical development should move beyond maximally tolerated dose determination alone. Drug exposure, intratumoral target engagement, DNA-damage pharmacodynamics, ctDNA kinetics, radiographic response, and resistance evolution should be evaluated as sequential but distinct components of the pharmacological response. Early-stage trials should therefore incorporate paired tumor biopsies where feasible, direct assessment of WRN engagement or occupancy, validated DNA-damage markers, longitudinal ctDNA sampling, and prospective sequencing for resistance-associated WRN alterations.

WRN inhibitors remain investigational and should not currently be considered standard therapy. Randomized efficacy data are unavailable, no validated companion diagnostic beyond MSI/dMMR enrichment has been established, and the durability of response and resistance spectrum remain incompletely defined. Prospective studies will need to determine whether structurally orthogonal inhibitors retain activity after on-target resistance and whether replication-stress or immune combinations provide sufficient additional benefit to justify their added toxicity.

## 8 Perspectives and conclusion

WRN-directed therapy exploits a well-established synthetic-lethal vulnerability created by mismatch-repair deficiency, but MSI-H/dMMR status should be considered an enrichment biomarker rather than a complete predictor of functional WRN dependency. The strongest current evidence—particularly for TA-repeat burden, pharmacological sensitivity, organoid dependency, and acquired resistance—derives from colorectal cancer. We therefore propose an evidence-graded translational framework that separates pretreatment predictors of biological dependency from target-engagement and DNA-damage pharmacodynamics, early treatment-response biomarkers, and resistance-monitoring markers.

Prospective studies should determine whether baseline TA-expansion burden predicts downstream DNA-damage and ctDNA responses at comparable intratumoral WRN occupancy, while simultaneously monitoring treatment-emergent WRN mutations and altered WRN abundance. This framework does not redefine quantitative WRN dependency as a new biological concept; rather, it organizes established biology and emerging pharmacology into experimentally testable clinical hypotheses. Extension of these principles to gastric, gastroesophageal, pancreatic, biliary, and other MSI-H gastrointestinal malignancies will require disease-specific functional and clinical validation.
